# Three-Dimensional Ultrasound for Physical and Virtual Fetal Heart Models: Current Status and Future Perspectives

**DOI:** 10.3390/jcm13247605

**Published:** 2024-12-13

**Authors:** Nathalie Jeanne Bravo-Valenzuela, Marcela Castro Giffoni, Caroline de Oliveira Nieblas, Heron Werner, Gabriele Tonni, Roberta Granese, Luis Flávio Gonçalves, Edward Araujo Júnior

**Affiliations:** 1 Department of Pediatrics, Pediatric Cardiology, School of Medicine, Federal University of Rio de Janeiro (UFRJ), Rio de Janeiro 21941-901, RJ, Brazil; njmbravo@icloud.com; 2 Department of Fetal Medicine, Biodesign Laboratory DASA/PUC, Rio de Janeiro 22453-900, RJ, Brazil; mcastrogiffoni@hotmail.com (M.C.G.); heron.werner@gmail.com (H.W.); 3 Discipline of Woman Health, Municipal University of São Caetano do Sul (USCS), São Caetano do Sul 09521-160, SP, Brazil; caroline.nieblas@uscsonline.com.br (C.d.O.N.); araujojred@terra.com.br (E.A.J.); 4 Department of Obstetrics and Neonatology, Istituto di Ricovero e Cura a Carattere Scientifico (IRCCS), AUSL Reggio Emilia, 42122 Reggio Emilia, Italy; tonnigabriele59@gmail.com; 5 Department of Biomedical and Dental Sciences and Morphofunctional Imaging, “G. Martino” University Hospital, 98100 Messina, Italy; 6 Departments of Radiology and Child Health, University of Arizona College of Medicine, Phoenix, AZ 85016, USA; luflagoncalves@gmail.com; 7 Department of Obstetrics, Paulista School of Medicine, Federal University of São Paulo (EPM-UNIFESP), São Paulo 04023-062, SP, Brazil

**Keywords:** fetal heart, congenital heart disease, ultrasonography, three-dimensional ultrasound, spatiotemporal image correlation, fetal intelligent navigation echocardiography, artificial intelligence

## Abstract

Congenital heart defects (CHDs) are the most common congenital defect, occurring in approximately 1 in 100 live births and being a leading cause of perinatal morbidity and mortality. Of note, approximately 25% of these defects are classified as critical, requiring immediate postnatal care by pediatric cardiology and neonatal cardiac surgery teams. Consequently, early and accurate diagnosis of CHD is key to proper prenatal and postnatal monitoring in a tertiary care setting. In this scenario, fetal echocardiography is considered the gold standard imaging ultrasound method for the diagnosis of CHD. However, the availability of this examination in clinical practice remains limited due to the need for a qualified specialist in pediatric cardiology. Moreover, in light of the relatively low prevalence of CHD among at-risk populations (approximately 10%), ultrasound cardiac screening for potential cardiac anomalies during routine second-trimester obstetric ultrasound scans represents a pivotal aspect of diagnosing CHD. In order to maximize the accuracy of CHD diagnoses, the views of the ventricular outflow tract and the superior mediastinum were added to the four-chamber view of the fetal heart for routine ultrasound screening according to international guidelines. In this context, four-dimensional spatio-temporal image correlation software (STIC) was developed in the early 2000s. Some of the advantages of STIC in fetal cardiac evaluation include the enrichment of anatomical details of fetal cardiac images in the absence of the pregnant woman and the ability to send volumes for analysis by an expert in fetal cardiology by an internet link. Sequentially, new technologies have been developed, such as fetal intelligent navigation echocardiography (FINE), also known as “5D heart”, in which the nine fetal cardiac views recommended during a fetal echocardiogram are automatically generated from the acquisition of a cardiac volume. Furthermore, artificial intelligence (AI) has recently emerged as a promising technological innovation, offering the potential to warn of possible cardiac anomalies and thus increase the ability of non-cardiology specialists to diagnose CHD. In the early 2010s, the advent of 3D reconstruction software combined with high-definition printers enabled the virtual and 3D physical reconstruction of the fetal heart. The 3D physical models may improve parental counseling of fetal CHD, maternal–fetal interaction in cases of blind pregnant women, and interactive discussions among multidisciplinary health teams. In addition, the 3D physical and virtual models can be an useful tool for teaching cardiovascular anatomy and to optimize surgical planning, enabling simulation rooms for surgical procedures. Therefore, in this review, the authors discuss advanced image technologies that may optimize prenatal diagnoses of CHDs.

## 1. Introduction

Congenital heart defects (CHDs) are the most frequent congenital malformations, with an incidence of approximately 6–12:1000 live births [1,2,3,4]. Concerning mortality due to congenital defects, it is important to bear in mind that this is one of the main causes of perinatal mortality [5]. However, the rate of prenatal diagnoses of CHDs remains low. It is known that more than 25% of CHDs are classified as critical, which means that these cardiac defects will require immediate care in the postpartum period by pediatric cardiology teams and surgery or a neonatal cardiac surgical procedure [6]. Therefore, early and accurate diagnosis of CHD is essential for proper prenatal and birth follow-up in a tertiary service.

The gold standard method for diagnosing CHD is fetal echocardiography [7]. This examination is carried out in few centers due to the need for trained professionals and high-tech equipment. Furthermore, since only 10% of CHDs come from high-risk groups, screening during the second trimester ultrasound scan is crucial. Currently, the addition of the ventricular outflow tracts and the superior mediastinum (three vessels and three vessels and trachea) views to the four-chamber view has improved the accuracy of screening for CHDs by almost 90% [7,8]. Despite the potential benefits of echocardiography in diagnosing CHDs, the requirement for a qualified professional and high-tech equipment currently precludes its routine implementation in clinical practice. Accordingly, the majority of international guidelines advocate for the referral of pregnant women for fetal echocardiography, contingent upon the risk profile of the population in light of the available evidence [9].

The advent of three-dimensional (3D) ultrasound in the early 1990s, and in particular, four-dimensional (4D) spatio-temporal image correlation (STIC) software in the early 2000s, marked a significant turning point in the field of fetal imaging. This has enabled significant technological advancement in fetal heart assessment, particularly in the identification of atrioventricular and semilunar valve regions, as well as the delineation of interatrial and interventricular septum areas and volumes, cardiac chambers, and ventricular walls [10,11,12,13,14]. The advantages of STIC in fetal cardiac assessment include a reduced dependence on the operator’s experience in obtaining diagnostic views, a shorter examination time with the analysis of volumes conducted in the absence of the patient, and the capacity to transmit volumes for analysis to reference centers in fetal cardiology via an internet link [15]. It is possible to “navigate” within the cardiac structures by moving from one plane to another, thus enabling the cardiac anatomy to be detailed and diagnoses regarding cardiac malformations to be clarified [16,17,18].

In the early 2010s, the advent of 3D reconstruction software, coupled with the advent of high-definition printers, enabled the virtual and physical reconstruction of fetuses and the virtual navigation of fetal cavities using 3D ultrasound and magnetic resonance imaging (MRI) data. This has facilitated realistic visualizations of structures, such as the upper airways in cases of cervical tumors, the urinary tract in cases of lower obstructions, and central nervous system malformations [19,20,21]. Furthermore, the use of physical 3D models has facilitated enhanced maternal–fetal interaction in cases of blind pregnancies, as well as more comprehensive parental understanding of fetal pathologies and interactive discussions among multidisciplinary medical teams [22,23]. With regard to the fetal heart, due to its specific aspect of movement, MRI data acquisition is still of low resolution, and 3D ultrasound using STIC software is still the preferred method. 

The 3D physical model realistically reconstructs the 3D ultrasound image, providing a better understanding of fetal heart disease for parents [24,25]. Most CHDs require immediate care in the postpartum period by a team of specialists, so an early and accurate diagnosis permits the formulation of an appropriate birth plan and, in cases of critical heart disease, facilitates the referral of the pregnant woman to a center with specialized cardiac team resources for prenatal care and scheduled delivery. This approach has the potential to reduce morbidity and mortality. The utilization of physical and virtual 3D reconstructions facilitates comprehensive visualization of cardiac anatomy and morphology, facilitating interactive discourse among the multidisciplinary team (obstetricians, neonatologists, pediatric cardiologists, cardiac surgeons, and specialist nurses) and enhanced comprehension of the anomaly by the parents [26,27,28].

Despite an increase in the number of ultrasound views included in the screening process for CHDs, the prenatal diagnosis of cardiac malformations remains inadequate. This is due to the limited access to high-quality ultrasound images and the interpretation of images obtained by non-specialists, particularly in cases of high complexity. The recent developments in fetal heart imaging technologies and artificial intelligence (AI) have the potential to enhance the prenatal detection of CHDs [29,30]. This review article provides an overview of several of these imaging technologies.

## 2. Three-Dimensional Ultrasound and Spatio-Temporal Image Correlation

The advent of fetal echocardiography approximately forty years ago has facilitated the study of embryonic development, morphology, biometry, and fetal heart function. Since 2003, 4D ultrasound with spatio-temporal image correlation (STIC) has enabled detailed digital assessment of the fetal heart. STIC technology, initially described by De Vore et al. [10], enables the acquisition of cardiac volumes using a volumetric transducer over a single scan period of 7.5 to 15 s, with the acquisition of 150 images per second. The reconstruction of these images in 3D, correlated to time (STIC), allows the image of the fetal heart in motion to be presented with high definition (cineloop). This technology allows for navigation through the valves, cavities, and vessels of the fetal heart, thereby facilitating an anatomical and functional detailed examination of the fetal heart [10,31]. Although this methodology is already familiar to practitioners, it is important to reiterate that to obtain high-quality cardiac volumes from the images used for the study of the fetal heart with STIC, it is recommended that the fetal spine is positioned at 6 o’clock (posterior dorsum) with an opening angle between 20° and 40° [32]. This is because unfavorable fetal statics, such as an anterior fetal dorsum and fetal movement during examination, can limit the accuracy of the image and pose a challenge to this technology, in a similar manner to two-dimensional (2D) ultrasound [33]. 

Furthermore, the assessment of cardiac volumes by STIC may be conducted with or without the patient (offline), as well as via the internet to tertiary centers (tele-STIC). Consequently, this technology has the potential to maximize cardiac screening by enabling remote access to fetal heart experts, thereby reducing costs and shortening distances. Fetal telemedicine has shown that it can improve prenatal parental counseling and cardiac screening by tele-medicine assisted or via a telemedicine link [34,35].

In this scenario, it is essential to highlight that 3D/4D spectral tissue imaging by reproducing a complete cardiac cycle allows the examiner in the multiplanar and rendering modes to select the optimal image quality and observe detailed morphological and functional aspects of the fetal heart [36,37]. Additionally, the examiner has the option of transmitting the selected images to the experts via the internet and being viable for obstetric ultrasound cardiac screening programs even by assisted ultrasound scanning of the fetal heart [34,35,38,39].

**Figure 1:**
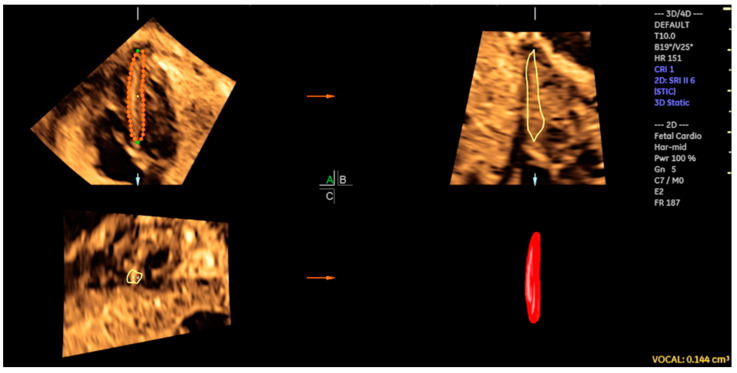
Measurements of interventricular septum volume (IVS) using 3D ultrasound with STIC and virtual organ computer-aided analysis (VOCAL) in a fetus from a diabetic mother at 25 weeks of gestation. IVS volume = 0.144 cm^3^.

**Figure 2:**
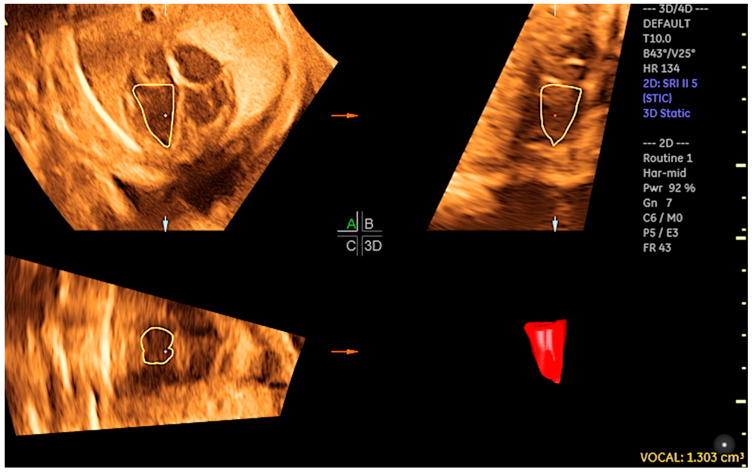
Left ventricle diastolic volume using STIC with virtual organ computer-aided analysis (VOCAL) in a fetus at 30 weeks of gestation. LV volume = 1.3 cm^3^.

**Figure 3:**
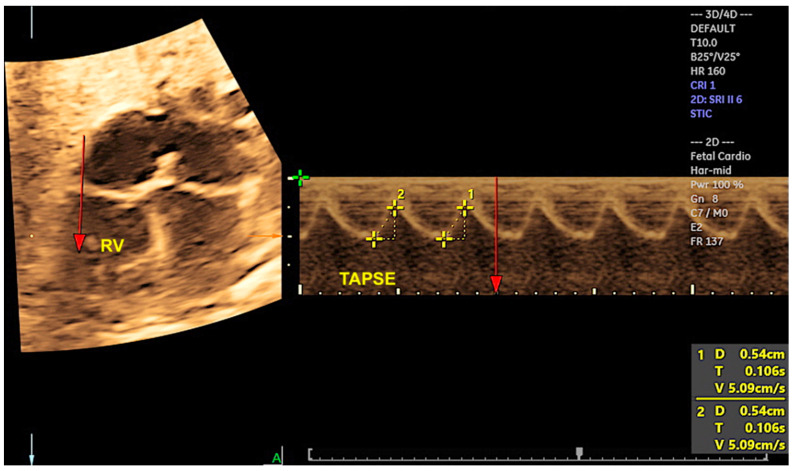
Evaluation of the tricuspid annular movement using fetal STIC-M (5.4 mm). TAPSE: tricuspid annular plane systolic excursion; RV: right ventricle.

**Figure 4:**
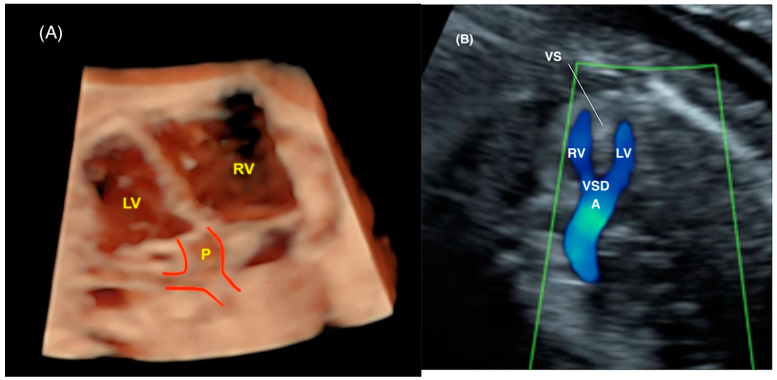
Three-dimensional ultrasound with STIC: (**A**) HDlive mode, providing a reconstruction of the left ventricular outflow tract in a case of transposition of the great arteries and (**B**) with color Doppler in a first-trimester fetus with tetralogy of Fallot. Observe the pulmonary artery (P) arising from the left ventricle (LV) in image (**A**) and the overriding of the aorta (**A**) in image (**B**). RV: right ventricle; LV: left ventricle; VSD: ventricular septal defect; IVS: ventricular septum.

**Figure 5:**
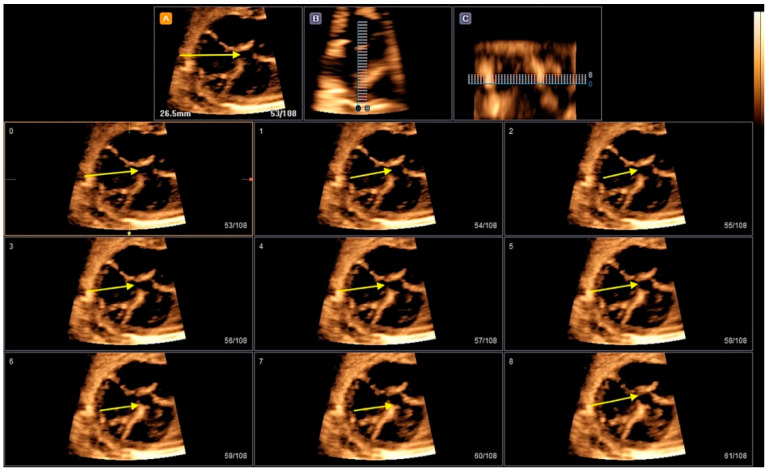
Tomographic ultrasound imaging (TUI) in the rendering mode enables the visualization of sequential axial planes in the case of inlet ventricular septal defect (VSD) (yellow arrows).

**Figure 6:**
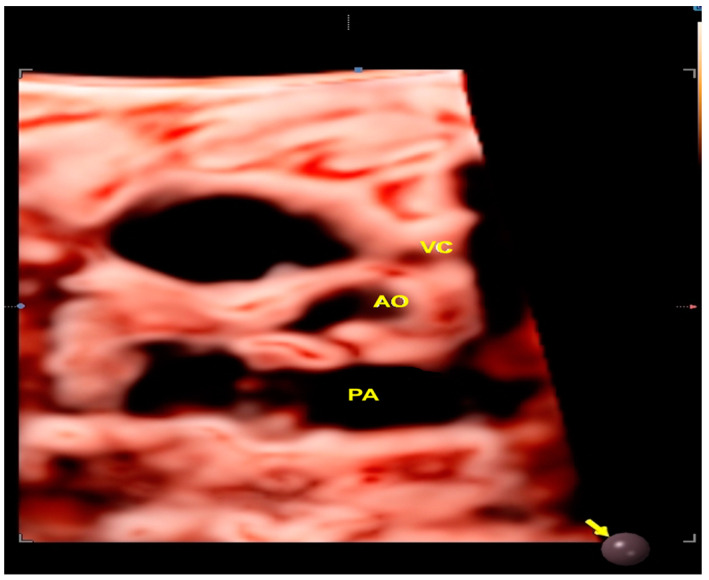
STIC with HDlive Silhouette mode in a case of coarctation of aorta. Note the discrepancy of the great arteries due to the small aorta. AO: aorta; PA: pulmonary artery; VC: superior vena cava.

By adjusting the brightness and color, STIC in rendering mode facilitates enhanced image clarity, thus enabling the diagnoses of structural CHDs, the measurement of the area and volume of various fetal heart structures, including the interventricular septum, valves, and papillary muscles (Figure 1), and the assessment of cardiac function in fetuses at risk of heart failure (Figure 2) [14,40,41]. STIC technology is capable of reconstructing moving images and displaying them in a variety of modes, including STIC-M, inversion mode, Doppler (color Doppler/HDlive flow), B-flow imaging, tomographic ultrasound imaging (TUI), and HDlive Silhouette (Figure 3, Figure 4, Figure 5 and Figure 6) [42,43,44,45]. 

Advanced technologies have the potential to elucidate complex CHDs, such as anomalous venous return, vascular rings, heterotaxy syndromes, and conotruncal anomalies. These imaging techniques can provide a more realistic appearance of the cardiac structures, which is an invaluable asset in the diagnosis and management of these conditions [46,47,48,49]. An accurate fetal cardiac diagnosis will have a significant impact on a number of medical areas, including decision-making and/or cardiac surgical procedures in utero or postnatally, improved birth planning, parental counseling, and, in countries where it is permitted by law, the decision to terminate pregnancies in a safer and less traumatic manner [9,33].

Nonetheless, the availability of advanced technology in primary care units is also a real limitation to routine obstetric practice. Training professionals in cardiac screening programs using 2D US technology is vital for the initial diagnosis of congenital heart disease in non-tertiary hospitals. Table 1 illustrates the clinical applications, advantages, and limitations of the main US technologies. 

### 2.1. Anomalous Venous Return

**Figure 7:**
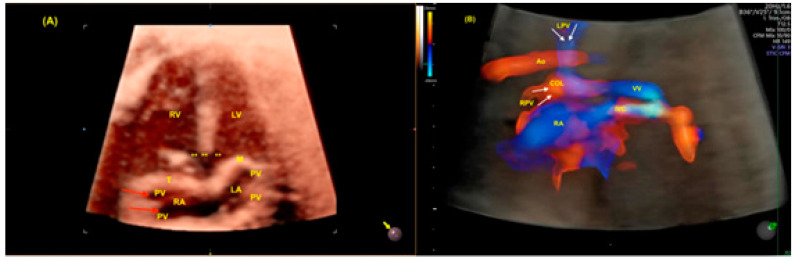
(**A**) Three-dimensional ultrasound with Surface Realistic Vue (SRV) imaging in a case of partial anomalous pulmonary vein return with a ventricular septal defect (VSD). Note that 2 of the pulmonary veins return to the right atrium (red arrows). Virtual light source position, 10 o’clock. (**B**) STIC with color Doppler of a case of total anomalous pulmonary vein return (infradiaphragmatic type). The right (RPV) and left pulmonary veins (LPVs) drain (white arrows) into a collecting vein (COL) and subsequently into a vertical vein (VV), which achieves the right atrium (RA) via the inferior vena cava (IVC). LV: left ventricle; LA: left atrium; RA: right atrium; RV: right ventricle; ** VSD: ventricular septal defect; PV: pulmonary vein; T: tricuspid valve; M: mitral valve.

**Figure 8:**
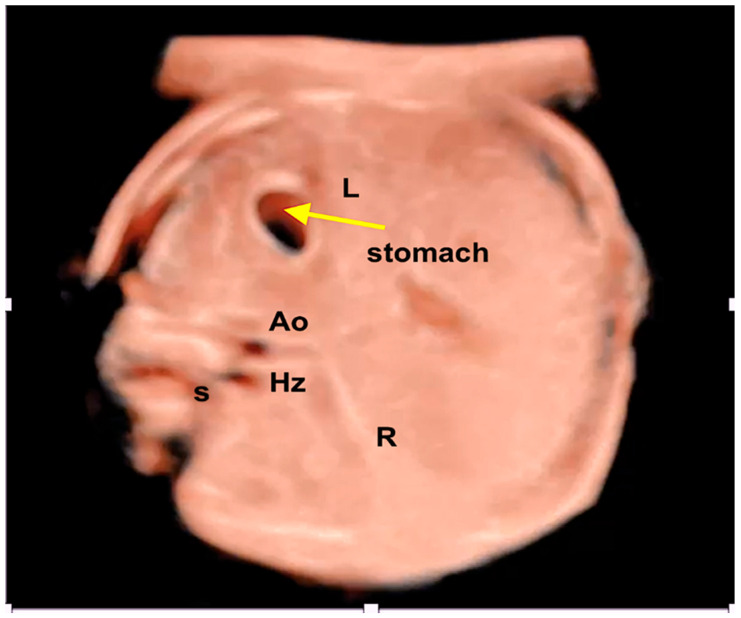
Three-dimensional ultrasound with STIC and HDlive mode in a case of left heterotaxy. Observe that the venous vessel (hemiazygos) is located posteriorly (near to the fetal spine) to the arterial vessel (aorta) at the upper abdomen view. Ao; aorta; Hz: hemiazygos vein; L: fetal left side; R: fetal right side.

Anomalous pulmonary venous return, in either its total or partial forms (Figure 7), is frequently misdiagnosed during the prenatal period. In this scenario, advanced fetal cardiovascular imaging technologies are useful tools for providing high-quality images with greater definition of the venous vessel walls and, consequently, of the drainage site as well as the points of obstruction. Underdiagnosis of total anomalous pulmonary venous return (TAPVR) affects postnatal outcomes, especially in obstructive forms (critical TAPVR), in which planned delivery and perinatal management are crucial to providing better outcomes [47,50,51]. Similarly, some umbilical vein anomalies (such as the agenesis of the ductus venosus and the persistence of the right umbilical vein) and systemic venous return anomalies (such as the agenesis of the portal vein and the interruption of the inferior vena cava) can be more accurately identified by 3D high-definition Doppler ultrasound. One of the most frequently encountered anomalies of the inferior vena cava (IVC) is the interruption of its intrahepatic segment at the suprarenal level. In such cases, the IVC flow typically returns to the right atrium via the azygos vein (or hemiazygos vein), which is associated with left heterotaxy or left atrial isomerism (also known as two morphologically left atria) (Figure 8) [46,52].

**Figure 9:**
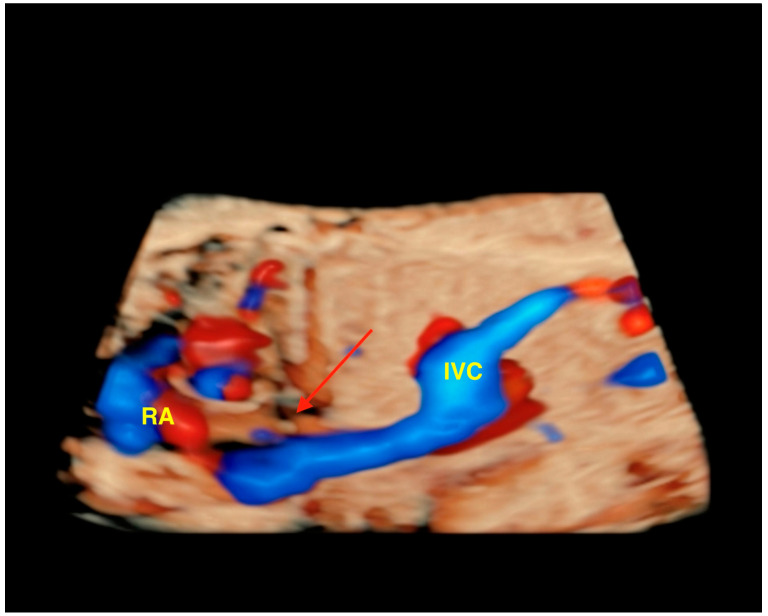
Extra-hepatic form of agenesis of ductus venosus using three-dimensional ultrasound with STIC. Note the high-resolution color Doppler showing the absence of flow through the DV (red arrow). In this case, the umbilical vein drains into the RA via the inferior vena cava. IVC: inferior vena cava; RA: right atrium.

In general, persistence of the right umbilical vein (PRUV) is a benign anatomic variant; however, it could be associated with cardiac and extra cardiac anomalies (trisomy 18, monosomy X, genitourinary and central nervous system anomalies, septal and conotruncal anomalies) [53,54]. Extra-hepatic forms of PRUV are associated with agenesis of ductus venosus (ADV) and a poor prognosis [55]. Indeed, ADV is associated with cardiac and extracardiac malformations, requiring a rigorous examination of the fetal anatomy and comprehensive testing for chromosomal anomalies, including trisomies 13, 21, 18, and monosomy X. The extrahepatic form of ADV is associated with a poorer prognosis and an elevated risk of hydrops [56,57]. In this scenario, the use of 3D color Doppler is beneficial for illustrating the spatial course of the vessels and the absence of flow through the DV (Figure 9). This can assist in predicting prognosis, identifying associated malformations, and providing parental guidance.

### 2.2. Transposition of the Great Arteries (TGA)

**Figure 10:**
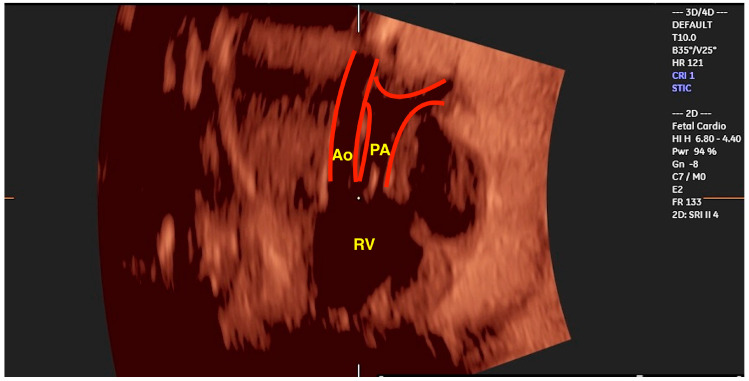
Three-dimensional ultrasound with STIC enabling the reconstruction of the ventricular outflow tracts in a case of double-outlet right vetricle (“Taussig-Bing” anomaly). Note the great arteries arising from the right ventricle (RV) in a parallel relationship. Ao: aorta; PA: pulmonary artery.

**Figure 11:**
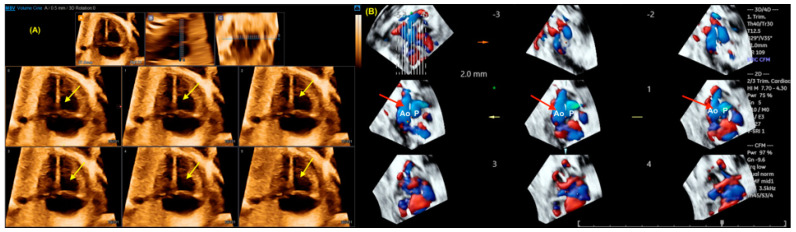
Tomographic ultrasound imaging (TUI) in the rendering mode in a case of tetralogy of Fallot and in (**B**) double outflow of the right ventricle (DORV). The right ventricle hypertrophy (yellow arrows) could be observed using this technology (**A**). Note the great arteries in a parallel relationship (red arrows) in a fetus with Taussig–Bing DORV using color Doppler (**B**). DORV: double outflow of right ventricle; Ao; aorta; P: pulmonary artery.

**Figure 12:**
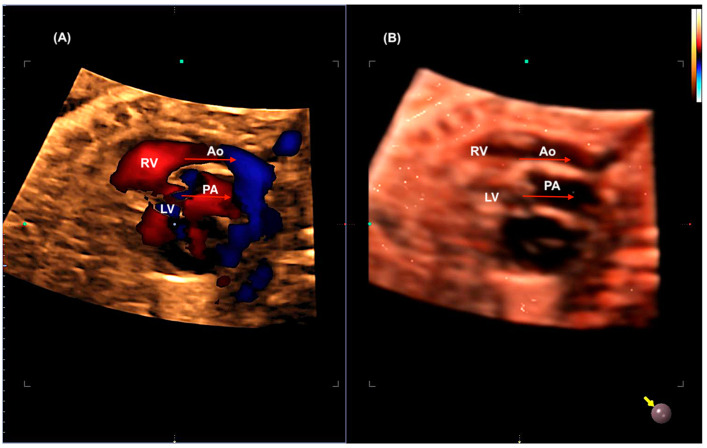
The reconstruction of the ventricular outflow tracts in a case of transposition of the great arteries (TGA) using STIC with color Doppler (**A**) and HDlive Silhouette. In image (**A**), it is evident that the aorta (Ao) arises from the right ventricle (RV). In image (**B**), the pulmonary artery (PA) is unequivocally identified as originating from the left ventricle (LV). The two arteries are observed to be in a parallel relationship (red arrows), with the aorta located anteriorly to the PA.

**Figure 13:**
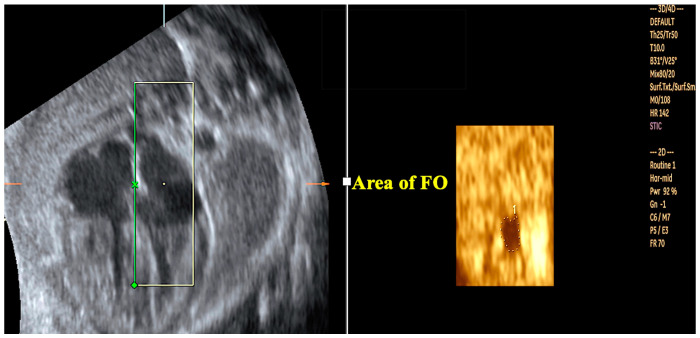
Three-dimensional ultrasound with STIC in the rendering mode: the measurement of the area of the foramen ovale (FO) was obtained from the four-chamber view of the fetal heart in which the ROI (green line) is the flap of the FO. ROI: region of interest.

The advantages of 3D/4D ultrasound in the diagnosis of conotruncal anomalies, including simple transposition of the great arteries (TGA), aortic arch interruption, and double-outlet right vetricle (“Taussig-Bing” anomaly), have been well-documented in the literature (Figure 10 and Figure 11) [58,59,60,61]. The automatic reconstruction of the outflow tracts from a 4D cardiac volume acquired from a standard four-chamber view facilitates the enhancement of the images of the ventricular outflow tracts, thereby improving the diagnostic accuracy. Moreover, TUI, inversion mode, HDlive Silhouette, HDlive flow, MV-Flow, LuminiFlow, and other advanced imaging technologies utilizing 3D ultrasound have the capacity to enhance visualization of the outflow tracts, thereby facilitating a more accurate diagnosis of TGA (Figure 12). These tools allow for a more efficient examination and facilitate the reconstruction of the great arteries [58,59,60,61]. In cases of TGA with progression to restriction of the foramen ovale (FO), it may be necessary to perform an in utero opening of the FO and/or urgent postnatal ballon atrioseptostomy. Maximum velocity of the pulmonary vein (a wave), FO flap mobility, and FO diameter are important parameters to perform this evaluation [62]. Measurements of the area of the interatrial septum using 4D ultrasound with STIC have been performed in normal fetuses with the construction of reference curves; however, the applicability in fetuses with CHDs has not yet been validated (Figure 13) [63]. Future directions using these measurements in STIC to evaluate restrictive FO may be promising.

### 2.3. Ebstein’s Anomaly (EA) and Tricuspid Dysplasia (TD)

**Figure 14:**
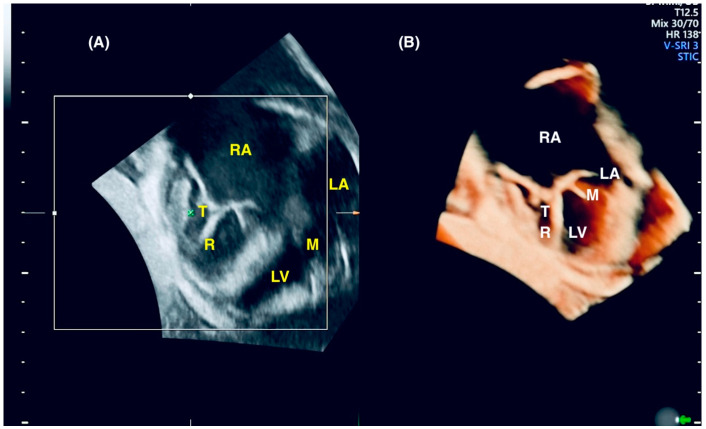
Three-dimensional with STIC in the rendering mode (**A**) and HDlive mode (**B**) of a fetus with Ebstein’s anomaly at 30 weeks of gestation. RA: right atrium; T: tricuspid valve; RV: right ventricle; LA: left atrium; M: mitral valve LV: left ventricle.

The most noteworthy distinction between Ebstein’s anomaly (EA) and tricuspid dysplasia (TD) pertains to the anatomical characteristics of the tricuspid valve. In EA, the tricuspid valve cusps are attached to the right ventricular myocardium, resulting in a displacement of the usual tricuspid valve plane and atrialization of the right ventricle. Conversely, in TD, the cusps are inserted normally at the level of the valve annulus, and regurgitation is caused by dysplasia with varying degrees of cusp deformation. Tricuspid regurgitation (TR) exists in EA and TD, and in fetuses with severe TR, an increase in right ventricle pressure is observed with pulmonary insufficiency and progression to circular shunting. In these cases, blood circulates through the arterial ductus instead of going to the fetal body and placenta (Figure 14). The maternal use of anti-inflammatory drugs (indomethacin and/or ibuprofen) between 20 and 34 weeks has been shown to reduce mortality in EA and TD [64].

### 2.4. Aortic and Ductal Arch Anomalies

**Figure 15:**
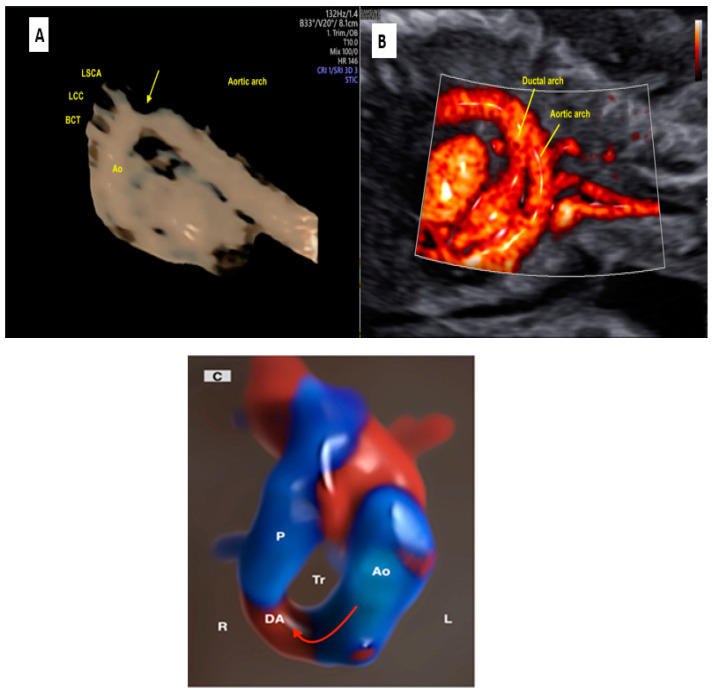
(**A**) Reconstruction of the aortic arch using STIC with the inversion mode in a case of coarctation of the aorta. Observe the narrowing of the aortic isthmus (yellow arrow). (**B**) Aortic and duct arch imaging in a fetus with a normal heart. (**B**) Sagittal view of a fetus with a normal heart showing the aortic and ductal arches using LumiFlow. (**C**) First-trimester imaging using HDFlow in a fetus with a right aortic arch (red arrow) and vascular ring (observe the vessels around the trachea). Ao: aorta; BCT: brachiocephalic trunk; LCC: left common carotid; LSCA: left subclavian artery; P: pulmonary artery; DA: ductus arteriosus; Tr: trachea; R: right side; L: left side.

Regarding vascular anomalies, STIC navigation with HDlive Silhouette, the post-processing inversion mode, high-resolution color Doppler such as HDlive flow, and LumiFlow techniques enable the detailed reconstruction of aortic and ductal arches with realistic images (Figure 15). In this scenario, anomalies of the laterality of the aortic arch, the origin of the vessels, and the vascular ring can be more easily clarified (Video S1) [65,66,67,68].

### 2.5. Cardiac Tumors

**Figure 16:**
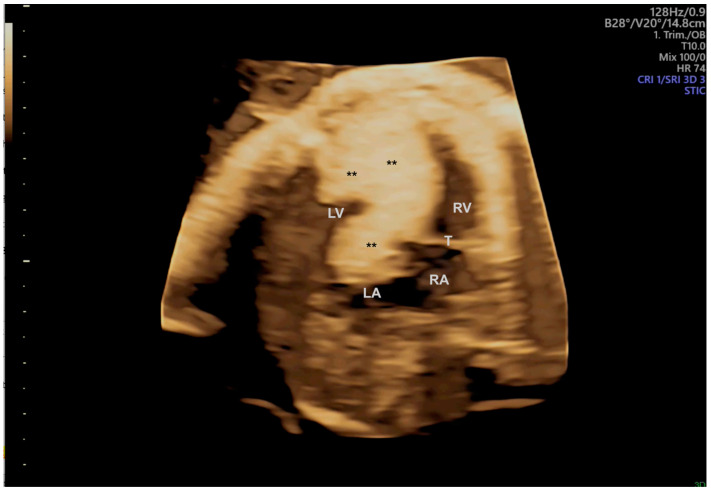
Large mass (**) in the ventricular septum and both ventricles, mainly in the left ventricle, in a case of rhabdomyomas with a reduction in the size of the masses after prenatal therapy with sirolimus. LV: left ventricle; LA: left atrium; RA: right atrium; RV: right ventricle; T: tricuspid valve.

Although cardiac tumors are rare, they can cause hemodynamic repercussions when there is obstruction to flow. Among fetal cardiac tumors, rhabdomyoma is the most prevalent. It is estimated that over 80% of rhabdomyomas are associated with tuberous sclerosis, which is linked to mutations in the TSC1 and TSC2 genes. These genes stimulate the production of hamartin and tuberin, thereby stimulating tumor proliferation. In cases of large masses with flow obstruction, myocardial dysfunction, and arrhythmias in fetuses that are not yet viable, some groups have been using mTOR inhibitors (sirolimus) from 23 to 35 weeks of gestation with success [69,70,71]. Teratomas represent 15% of fetal cardiac tumors and are most frequently intrapericardial. They have the potential to grow rapidly and may be associated with large pericardial effusions, which can result in fetal hydrops [72]. In such cases, mortality is high, and aspiration of the pericardial effusion in utero is a crucial procedure. In this scenario, 3D ultrasound is a particularly useful tool for identifying the type of tumor (Figure 16).

### 2.6. Cardiac Functional Evaluation

**Figure 17:**
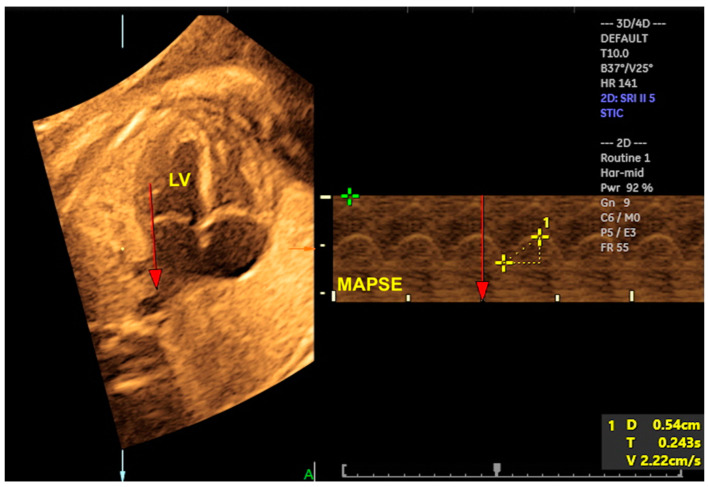
STIC-M enabling the measurement of mitral annular plane systolic excursion (MAPSE) (5.4 mm). LV: left ventricle.

**Figure 18:**
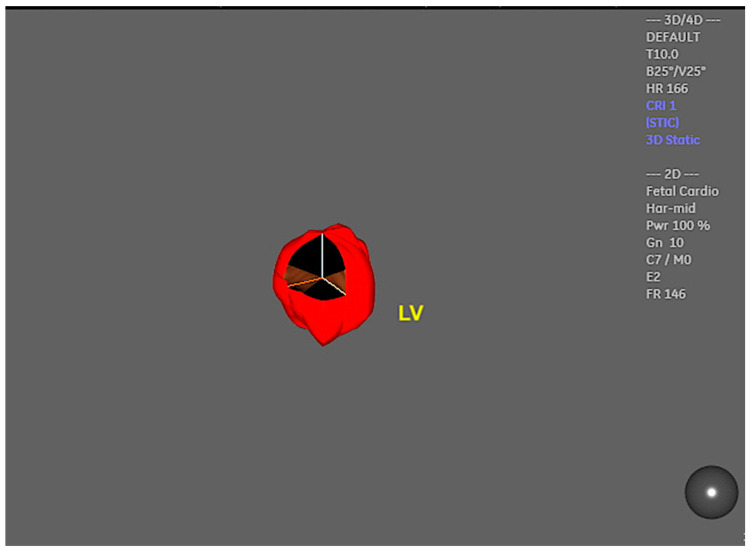
Three-dimensional reconstruction of the left ventricle (LV) using STIC with virtual organ computer-aided analysis (VOCAL) in a fetus at 22 weeks of gestation.

The cardiovascular score, which is based on a variety of parameters obtained from 2D Doppler ultrasound, enables the assessment of fetuses who are at risk of developing heart failure. Nevertheless, at the initial stages of myocardial dysfunction or even to provide detailed cardiac function assessment, 4D ultrasound with STIC can assist in the evaluation of STIC-M, tricuspid annular plane systolic excursion (TAPSE), and mitral annular plane systolic excursion (MAPSE), which are parameters that assess systolic dysfunction at an earlier stage than the filling fraction or the calculation of the ejection fraction by virtual organ computer-aided analysis (VOCAL) (Figure 17 and Figure 18/Videos S2 and S3). This can be beneficial in cases of diabetic fetuses and fetuses with vascular malformations such as Galen, tumors, and other conditions. Therefore, functional parameters (stroke volume, cardiac output, and ejection fraction) can be assessed using the STIC with VOCAL. Each ventricular volume is measured at the end of diastole (maximum volume) and at the end of systole (minimum volume). The caliper is positioned inside the basal and apical ventricular regions. After six sequential plans, manually delineated for each ventricle, VOCAL provides the chamber volume and reconstructs its 3D image. Finally, the stroke volume, cardiac output, and ventricular ejection fraction values are calculated for each ventricle [73,74,75,76,77]. 

## 3. Fetal Intelligent Navigation Echocardiography (FINE) and Artificial Intelligence 

**Figure 19:**
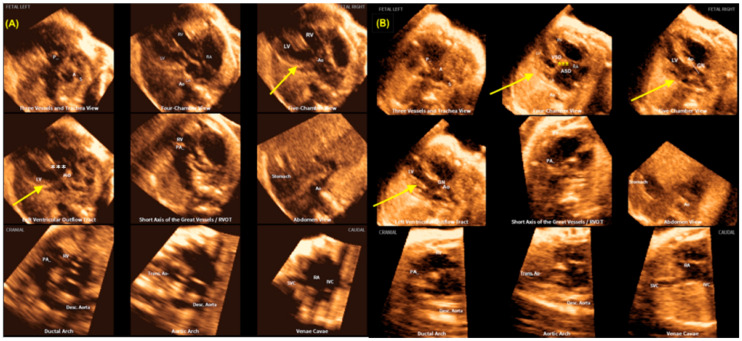
FINE navigation (known as “5D heart”) in (**A**) a case of a malalignment type of ventricular septal defect (***, yellow arrows) and in (**B**) a case of complete atrioventricular septal defect (AVSD). In case (**A**), observe the overriding of the aorta (Ao). In case (**B**), observe that the four-chamber, the five-chamber, and LV outflow tract (LVOT) views (yellow arrows) draw attention to this diagnosis. *** Common AV valve; VSD: ventricular septal defect; ASD: primum atrial septal defect; GN: LVOT with a “goose neck” shape.

The fetal intelligent navigation echocardiography (FINE) technique, referred to as “5D heart”, comprises the automated reconstruction of the nine planes that have been standardized by fetal echocardiography. The software utilizes intelligent navigation to direct the examiner in the marking of seven points (the descending aorta, crux cordis, pulmonary valve, superior vena cava, and transverse aorta) and subsequently, the nine fetal echocardiography views are reproduced in sequence [78,79,80]. This is achieved by marking strategic cardiac anatomical points from the four-chamber view of the fetal heart (Figure 19). The diagnostic views and VIS-Assistance videoclips can be transmitted to experts in fetal cardiology [78,79,80,81].

**Figure 20:**
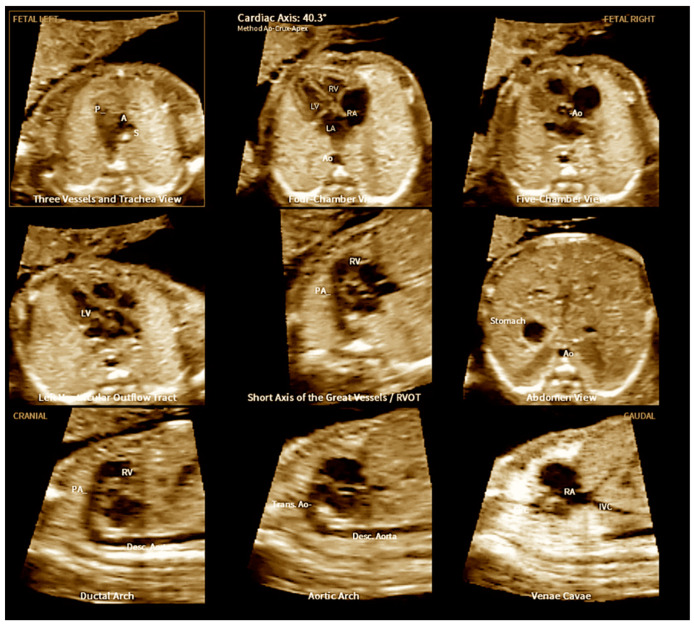
Automatic measurement of the fetal the cardiac axis (40.3º) using artificial intelligence (“Learning Machine”) in a normal heart using fetal intelligent navigation echocardiography (FINE), also known as “5D Heart”. LV: left ventricle; LA: left atrium; RA: right atrium; RV: right ventricle; A or Ao: aorta; P or PA: pulmonary artery; S: superior vena cava; IVC: inferior vena cava; Desc: descending; Trans: transverse.

Automatic applications, including structure identification, automated measurements, and providing alerts on probable diagnoses, have been added to FINE technology. This recent AI software, designated as HeartAssist^TM^ (Samsung Co., Seoul, South Korea) is capable of recognizing fetal heart structures, performing automatic (anatomical and functional) measurements, reducing examination time, and minimizing measurement errors related to operator–examinator variations (Figure 20) [82,83,84]. 

The evidence concerning the potential benefits of AI in fetal cardiac ultrasound assessment, as demonstrated by HeartAssist^TM^, is encouraging. The current focus within the field of AI is the development of automated techniques for the detection of specific types of CHDs, such as hypoplastic left heart syndrome, atrioventricular septal defect, and tetralogy of Fallot. For example, in tetralogy of Fallot, fetuses with a pulmonary valve Z-score of less than −5.0 and a Z-score difference between the pulmonary and aortic valves of >5 have an increased risk of pulmonary flow dependence on the ductal arch flow [85]. The measurements can be plotted in fetal Z-scores against the gestational age, biparietal diameter, and femur length, helping to categorize the diameters of the pulmonary valve and arteries.

**Figure 21:**
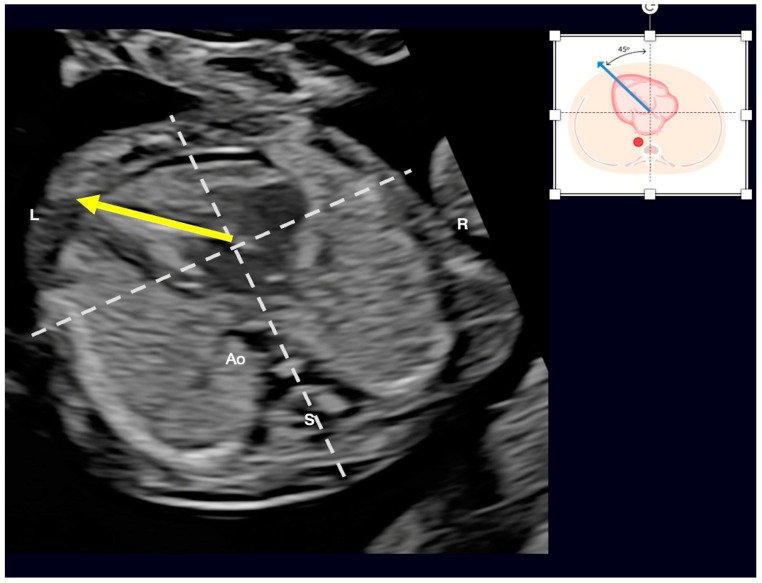
First-trimester measurement of the cardiac axis (45°) of a normal fetus (yellow arrow). L: left side; R:right side: Ao: aorta; S: spine.

Automating the measurements of cardiac structures using AI during the screening process provides an objective approach that can be performed in busy clinical environments, reduces operator variability, and extends fetal heart screening by alerting non-specialists to malformations. The HeartAssist^TM^ method is a potential method for automated fetal cardiac structure measurements. The automated measurements of the cardiac axis and some of the functional parameters using this software are feasible even at first-trimester ultrasonography cardiac evaluations (Figure 21). The establishment of reference curves of the automated Z-scores of the fetal heart obtained by AI represents an encouraging avenue of research, and the development of these automated techniques remains a work in progress [86].

## 4. Three-Dimensional Physical and Virtual Models of the Fetal Heart

**Figure 22:**
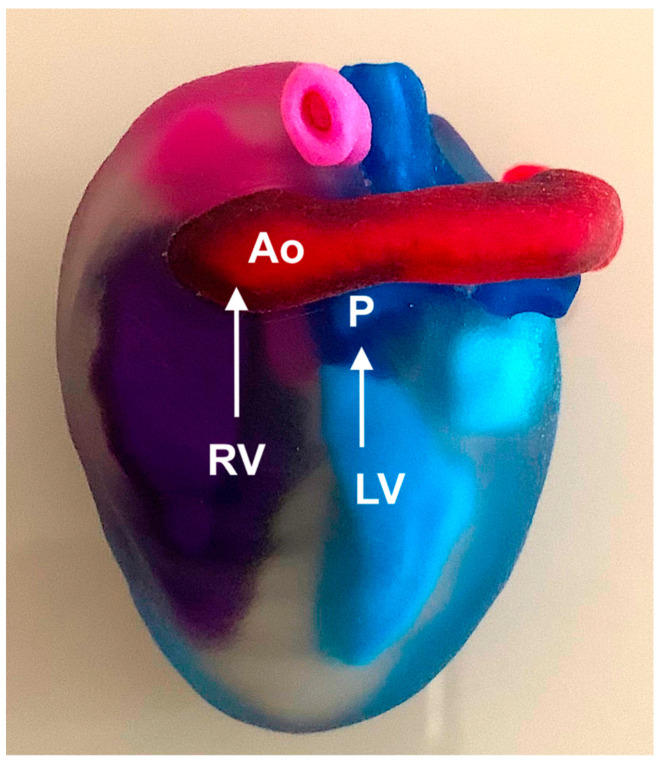
Three-dimensional physical model of a fetus with transposition of the great arteries (TGA). RV: right ventricle; Ao: aorta; LV: left ventricle; P: pulmonary artery.

**Figure 23:**
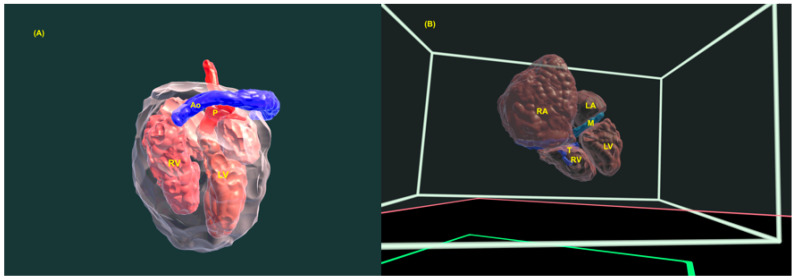
Three-dimensional virtual model of fetal heart in a fetus with transposition of the great arteries (TGA) (**A**) and in a fetus with Ebstein’s anomaly (**B**). RA: right atrium; RV: right ventricle; LA: left atrium; T: tricuspid valve; M: mitral valve; LV: left ventricle; Ao: aorta; P: pulmonary artery.

4D ultrasound with STIC software provides the acquisition of cardiac volumes in the multiplanar and rendering modes, enabling offline navigation through the structures of the fetal heart. Physical and virtual 3D reconstruction of the fetal heart can be created from 3D ultrasound volumes (Figure 22 and Figure 23). This technology facilitates a more complete understanding of the anatomical changes associated with CHDs and provides a platform for interactive discussions among the multidisciplinary medical team [87,88].

**Figure 24:**
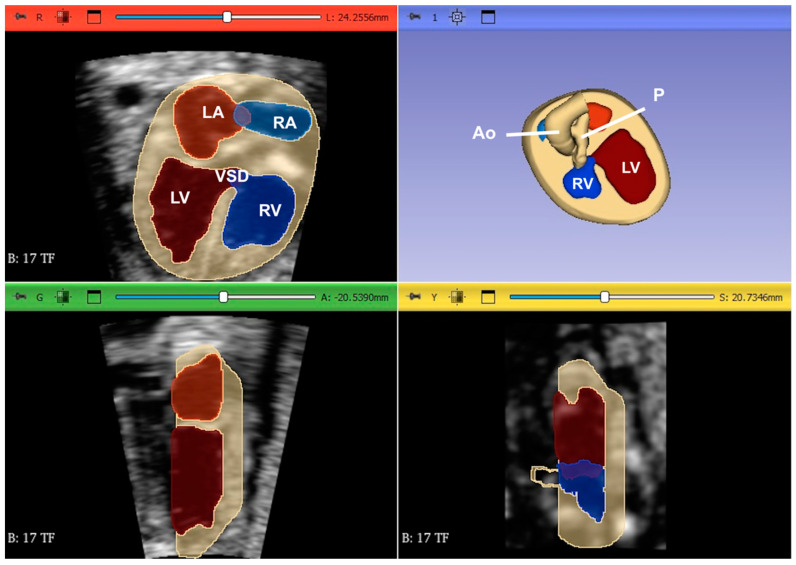
Following the acquisition of images of the fetal heart with tetralogy of Fallot from 3D ultrasound (heart volumes) using tools from Slicer 3D software (Birmingham, UK), the cardiac structures were segmented, with each cavity identified by a different color (right and left atrium, right and left ventricles, aorta, pulmonary artery, vena cava and pulmonary veins). Thereafter, a raw file format was generated. Based on the 3D data, physical 3D models of the fetal heart were printed using a 3D printer. Ao: aorta; LA: left atrium; P: pulmonary artery; RA: right atrium; LV: left ventricle; RV: right ventricle; VSD: ventricular septal defect.

After acquiring images of the fetal heart from 3D ultrasound (heart volumes), using tools from Slicer 3D (Birmingham, UK) software, different masks on each image were created in a sequence (Figure 24). Each mask corresponds to a particular structure. For example, a blue mask delineates a cavity or vessel within the fetus, thus defining the boundaries between structures. The program identifies all pixels in the sequence with a shade between the two values and selects them by creating an overlapping mask and assigning a color to each cardiac chamber or vessel. Once the segmentation was complete and the structures were properly separated into the masks, the 3D physical model was generated. From the 3D data generated, 3D physical models of the fetal heart were printed using a 3D printer [26]. 

In the process of creating virtual models of the fetal body, the 3D ultrasound and MRI images of the fetal heart were exported to a 3D virtual navigation software named Elucis (Elucis software, Realize Medical, Ottawa, ON, Canada). During the rendering process, the software calculates the lighting and shading effects according to the color and shape of the model, saving the images produced throughout the process (30 images for every second of movement). The aforementioned images were employed to structure the navigation video and saved as an MP4 video [28]. 

The creation of 3D physical models of CHDs can facilitate the enhancement of anatomical details and contribute to the accuracy of diagnostic procedures. In addition, the use of 3D physical models can facilitate parental counselling and may enhance therapeutic planning of cardiac diseases by a multidisciplinary team, as they provide a more comprehensive understanding of the CHD [89,90,91]. Moreover, these 3D resin models can be employed in classes of cardiac anatomy. Virtual navigation has been utilized in other medical specialties for surgical training, and in fetal cardiology, it represents a promising tool due to its capacity to facilitate the training and planning of postnatal surgical procedures in virtual simulation rooms [27].

The utilization of virtual simulation rooms facilitates the participation of experts and students from disparate geographical locations, thereby reducing the physical distances that would otherwise be traversed. In this way, the team can be trained in surgical procedures before actually performing them, and a more experienced medical team can assist remotely [92]. 

## 5. Fetal Cardiac Magnetic Resonance Imaging

Fetal cardiac MRI is still limited by high fetal heart rates and the small size of fetal heart structures. The time required for the examination is also a limitation for the pregnant woman. On the other hand, unfavorable fetal statics is a major limitation for ultrasound, but not for MRI [93]. 

**Figure 25:**
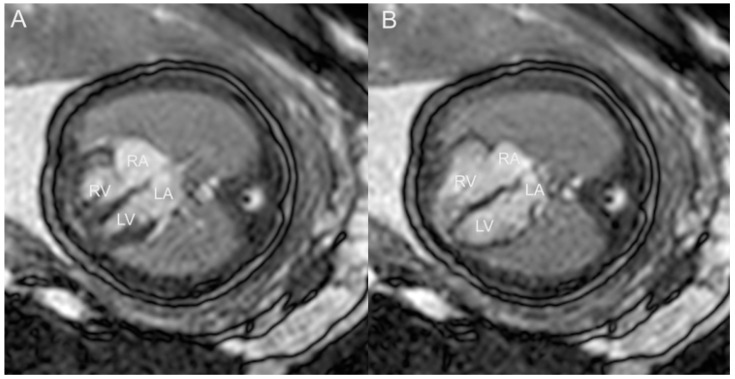
(**A**) Fetal cardiac MRI (fCMR) performed at 32 weeks and 5 days. Images were obtained at 1.5 T using a balanced turbo field echo (BTFE) sequence, gated with an MRI-compatible Doppler ultrasound (DUS) device (North Medical, Hamburg, Germany). Four-chamber view in systole (**A**) and diastole (**B**). LA: left atrium; LV: left ventricle; RA: right atrium; LA: left atrium.

MRI is also capable of non-invasively assessing oxygenation through the calculation of both oxygen saturation and the hematocrit of the blood within a vessel, utilizing the T1 and T2 relaxation times. Fetal cardiac MRI has yielded significant insights into the circulatory systems. Fetal cardiac MRI may be useful when conventional fetal echocardiography is inconclusive, especially in vascular malformations, such as vascular rings, aortic anomalies, and arteriovenous malformations (Galen vein aneurisms, coronary fistulas, and hemangiomas) [94,95,96]. Fast sequences, such as single-shot T2-weighted turbo spinecho (TSE) MR with half-Fourier reconstruction and steady-state free precession (SSFP), are the most used sequences for the assessment of fetal hearts [97]. Bright-blood SSFP sequences provide optimal contrast between the blood and myocardium cavities [98] (Figure 25). 

A study evaluated the clinical utility of fetal cardiac MRI in cases where conventional fetal echocardiography did not visualize all anatomic structures. Of a total of 31 fetuses, fetal cardiac MRI was helpful in the management and/or parental counseling in 26 cases (84%). For aortic arch anatomy, including signs of coarctation (20 cases), fetal cardiac MRI added diagnostic information in 16 cases (80%). In four fetuses with hypoplastic left heart syndrome (HLHS), fetal cardiac MRI helped delivery planning in three cases (75%) [99]. 

In a retrospective cohort study involving 431 singleton pregnancies (62 fetuses with CHDs and 269 healthy fetuses), MRI cardiac axes were measured in both the CHD and normal fetuses. The authors observed a good inter- and intraobserver reproducibility of the fetal cardiac axes measured by echocardiography and MRI [100]. In another study, the authors assessed the contribution of fetal cardiac MRI to identify cardiovascular anomalies in fetuses with CHDs. The authors identified 73 (60.3%) cardiovascular anomalies in 31 fetuses with CHDs. Fetal echocardiography was more sensitive for diagnosing cardiovascular anomalies compared to fetal MRI, but without significant differences [101].

**Figure 26:**
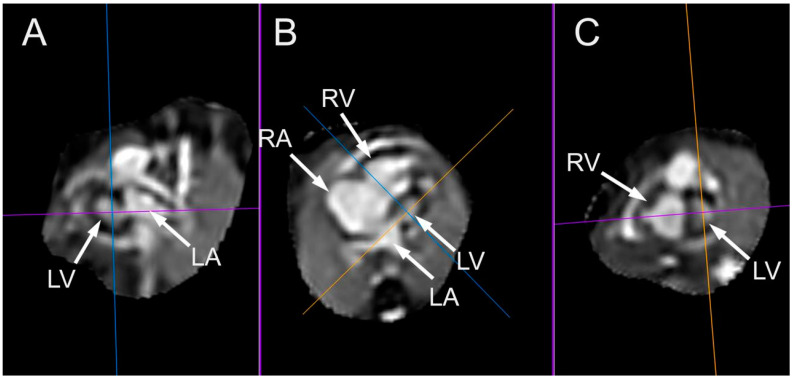
Multiplanar display images of a case of hypoplastic left heart syndrome examined at 32 weeks and 3 days. The images were acquired using a balanced turbo field echo (BTFE) sequence at 1.5 T. kt-sense acceleration was used during acquisition. The images were postprocessed using a super-resolution pipeline, resulting in an isovoxel 3D volume dataset. (**A**) Sagittal two-chamber view. (**B**) Four-chamber view. (**C**) Coronal short-axis view through the ventricles. LA: left atrium; LV: left ventricle; RA: right atrium; RV: right ventricle.

A cohort study involving 13 fetuses (24–36 weeks of gestation) with CHDs evaluated the image quality for the slice-to-volume reconstruction of 4D balanced steady-state free precession (bSSFP) imaging of the fetal heart. Two radiologists evaluated. the slice-to-volume reconstruction image quality on a scale from 0 to 4 using 11 categories based on a segmental approach to defining cardiac anatomy and pathology. In 11 out of 13 cases, the average radiologist score of image quality across all categories was ≥3.0 [102] (Figure 26).

## 6. Conclusions

The application of advanced imaging techniques facilitates the acquisition of images with greater clarity, enabling the delineation or clarification of anatomical structures and the navigation of cardiac structures and vessels in real time and offline. Furthermore, these techniques can assist in the screening of CHDs by non-specialists in fetal cardiology. In this scenario, the incorporation of advanced technology into the 2D ultrasound images of the fetal heart can enhance the prenatal detection of CHDs. Automated measurements of the anatomical and functional structures of the fetal heart can reduce operator-dependent discrepancies. In addition to measurements, AI can assist in the identification of potential cardiovascular alterations. The prospective applications of 3D physical and virtual models of fetal cardiac structures and functions are promising tools for the perinatal management of cardiovascular diseases.

## Figures and Tables

**Table 1:** Applications, advantages, and limitations of the 3DUS technologies.

Method	Clinical Applications and Advantages	Limitations
STIC	Acquisition of 150 images/s (cardiac volumes) Reconstruction of the cardiac volumes, elucidating complex CHD Off-line assessment, tele-medicine assistance Addition of other modalities: inversion mode, Doppler, TUI, HDlive, VOCAL	Availability of 3DUS machines with STIC software Unfavorable fetal statics Maternal obesity Fetal movements and maternal respiratory movements
HDlive and SRV	Detailed reconstruction of cardiac structures Realistic images	Availability of 3DUS machines with STIC software
HDFlow and HD Doppler modalities	Better resolution for margins of the vessels with less blooming Detail and sensitivity of blood flow	Availability of 3DUS machines with HDFlow and HD Doppler software
MVFlow, LumiFlow	Evaluation of the fetal microvasculature, especially the great arteries	Availability of 3DUS machines with MVFlow and LumiFlow software
FINE (“5D heart”)	Intelligent navigation Automated reconstruction of the 9 planes of fetal echocardiography Reduce examination time Tele-medicine assistance	Availability of 3DUS machines with FINE software Unfavorable fetal statics and maternal obesity
HeartAssist (AI software)	Recognize fetal heart structures Perform automatic measurements Reduce examination time Minimize measurement errors related to operator-examinator variations	Availability of 3DUS machines with HeartAssist software
3D physical models and VR	More comprehensive understanding of the cardiac anatomy (parental counselling, patient care, health professional education) Training and planning of surgical procedures in virtual simulation rooms	Cost of virtual navigation software and 3D printers

CHD: congenital heart defect; STIC: spatio-temporal image correlation; 3D: three-dimensional; 4D: four-dimensional; SRV: Surface Realistic Vue; HD: high-definition; US: ultrasound; FINE: fine intelligent navigation echocardiography; AI: artificial intelligence; TUI: tomographic ultrasound imaging; VOCAL: virtual organ computer-aided analysis; VR: virtual reality.

## Data Availability

The data presented in this study are available upon request from the corresponding author.

## Supplementary Materials

The following supporting information can be downloaded at https://www.mdpi.com/article/10.3390/jcm13247605/s1: Video S1. Sagittal view of aortic arch using STIC with Doppler in a case of coarctation of aorta. Observe the narrowing of the aortic isthmus; Video S2. How to obtain the measurement of mitral annular plane systolic excursion (MAPSE) using fetal STIC-M; Video S3. How to measure the left ventricle (LV) systolic volume using STIC with virtual organ computer-aided analysis (VOCAL). The caliper is positioned inside the basal and apical ventricular regions. After six sequential plans, manually delineated for LV, VOCAL provides the ventricular chamber volume. Indeed, the stroke volume (SV), cardiac output (CO), and ejection fraction (EF) can be calculated for each ventricle by the measurement of the ventricular volume at the end of diastole and at the end of systole; Video S4. Virtual navigation in a case of transposition of the great arteries.
